# Limitations of athlete-exposures as a construct for comparisons of injury rates by gender/sex: a narrative review

**DOI:** 10.1136/bjsports-2024-108812

**Published:** 2024-12-04

**Authors:** Ann Caroline Danielsen, Annika Gompers, Sheree Bekker, Sarah S. Richardson

**Affiliations:** 1 Social and Behavioral Sciences, Harvard T.H. Chan School of Public Health, Boston, Massachusetts, USA; 2 Department of Epidemiology, Emory University Rollins School of Public Health, Atlanta, Georgia, USA; 3 Department for Health, University of Bath, Bath, UK; 4 Department of the History of Science, Harvard University, Cambridge, Massachusetts, USA; 5 Committee on Degrees in Studies of Women, Gender, and Sexuality, Harvard University, Cambridge, Massachusetts, USA

**Keywords:** Anterior Cruciate Ligament, Athletic Injuries, Review, Women in sport, Public health

## Abstract

High rates of anterior cruciate ligament (ACL) injuries in girls’ and women’s sports have garnered significant attention from researchers, sport organisations and the media. Gender/sex disparities in ACL injury rates are often estimated using the construct of athlete-exposures (AEs), a widely used measure of exposure time in sports science and epidemiology that is defined as one athlete participating in one practice or competition. In this narrative review, we explain the limitations of AEs as a measure of exposure time and develop a series of conceptual critiques regarding the use of AEs for the purposes of comparing injury rates by gender/sex. We show that the differing training-to-match ratio and average team size between women and men—rooted in persistent gendered inequities in sports participation and professionalisation—may jeopardise the validity of using AEs for cross-gender comparisons and skew gender/sex disparities in ACL injury rates. To avoid bias, we invite researchers interested in gender/sex disparities in injury rates to collect finer-grained data including individual-level AEs disaggregated by training and competition, as well as to appropriately control for team size and training-to-match ratio at the data analysis stage. Any quantitative comparisons of injury rates should also thoroughly contextualise the limitations of AEs, including their inability to capture the potential qualitative differences between women’s and men’s training and sporting environments that may influence injury rates.

**WHAT IS ALREADY KNOWN**

**WHAT ARE THE NEW FINDINGS**

- AEs are an undertheorised and underscrutinised construct for comparisons across genders.
- AE as a construct may lead to inaccuracies in estimating gender/sex disparities in ACL and other injury risk, particularly due to systematic gender differences in training-to-match ratio and roster size.
- Researchers must consider whether the AEs that comprise the rate denominator for each group are not only quantitatively, but also qualitatively, comparable.

## Introduction

 Anterior cruciate ligament (ACL) injuries in girls’ and women’s sport have in recent years been described as an ‘epidemic’.^1^ Claims that women experience ACL injury at rates 2–10 times higher than men circulate widely and in forums as diverse as the sport and orthopaedic medicine literature,^2^,^4^ professional sport organisations^5^ and the media.^6 7^ One recent systematic review reported that women’s ACL injury rate in contact sports is threefold that of men, with 1.88 injuries per 10 000 athlete-exposures (AEs) among women compared with 0.87 per 10 000 AEs among men.^8^ While some scholars have noted the possible link between ACL injury and the gendered environment,^9^ injury research continues to characterise differences in ACL injury susceptibility as primarily due to innate sex-linked factors. According to these theories, the root causes of women’s greater susceptibility to ACL injury are found in (1) sex differences in musculoskeletal anatomy such as women’s increased ligament and joint laxity,^10 11^ narrower intercondylar notch^12^ or wider Q angle (measured as the angle formed between the pelvis and the kneecap),^13^,^16^ (2) the influence of the menstrual cycle^17^,^21^ or (3) a combination of the above. These mostly speculative hypotheses have been widely circulated and are increasingly shaping research programmes that investigate gender/sex disparities in ACL injury,^22 23^ as well as prevention and intervention programmes aimed at decreasing them.^24^

Incidence rates, such as those used to highlight disparities in the occurrence of ACL injury between women and men, are calculated by dividing the total number of injuries by the person-time at risk. Person-time is a measure of cumulative exposure time, *ie,* the total time that individuals in the study spent exercising, training or competing in games, matches or races. In sports injury research and sports epidemiology, person-time is commonly captured and reported using the field-specific construct of AEs.^25 26^ An AE is defined as one athlete participating in one competition or practice.^26^ Importantly, AEs are often used as the reference metric to which alternative measures of exposure time are converted when pooling ACL injury rates for women and men across different studies for the purposes of conducting comparisons (*eg*, in meta-analyses).^8 27 28^ While AEs have been criticised in the literature for the bias that they may introduce into calculations of injury rates by capturing aggregate rather than individual-level time at risk,^29^ the limitations of AEs in the context of estimating gender/sex disparities in injury occurrence have not been examined. Accurate measurement is crucial in order to understand and address ACL injury among women. By calling attention to social factors that are potentially missed in current epidemiologic metrics, we hope that our analysis contributes to rigorous measures and inferences in this vital area of women’s sports research.

This narrative review assesses the shortcomings of AEs for the purposes of generating valid and robust statistical inference on injury risk by gender/sex (box 1). First, we provide an overview of the emergence of AE as a construct and review its limitations. Second, we look at what is captured in calculations of AEs and query their suitability for conducting comparisons of injury rates by gender/sex. In particular, we illustrate how the different realities of women and men’s sporting contexts may threaten the comparability of crude AEs by gender. We argue that failing to consider gendered inequalities in training-to-match ratio and team size when computing AEs may artificially inflate apparent gender/sex disparities in ACL injury rates. Finally, we discuss the gendered contextual factors that may contribute to gender/sex disparities in injury rates that are not captured in AEs, and highlight how these may warrant consideration when interpreting injury rate comparisons by gender/sex. We also offer recommendations for increasing the precision, robustness and validity of AEs in the context of analysing gender/sex disparities.

Box 1 **Sex, gender and gender/sex**

Sex refers to the set of biological characteristics pertaining to an individual’s chromosomal, gonadal, hormonal and reproductive anatomy makeup. Notably, while sex is usually assumed to neatly divide the population into females and males, the substantial variability in combinations and phenotypes of sex-linked characteristics makes binary definitions of sex scientifically inaccurate and of limited usefulness for operationalisation in research.

Gender refers to individual identity, roles and behaviours, as well as the social structures and historical context that inequitably distribute power and resources across genders.

Gender/sex is ‘an umbrella term for both gender (socialization) and sex (biology, evolution) and *reflects social locations or identities where gender and sex cannot be easily or at all disentangled*’ (emphasis added).^80^ Throughout this narrative review, we choose to use this term when referring to disparities in ACL injury between women and men to highlight that the aetiology of such disparities is likely the result of a complex interplay between biological and social factors.

ACL, anterior cruciate ligament.

## Methods

This study used a narrative review methodology,^30^ through which we systematically reviewed the sports injury literature employing AEs and integrated studies examining gendered processes in sport. We began by assessing the evidence base on gender/sex disparities in ACL injuries measured through AEs, using the studies included in two recent systematic reviews as a foundational reference.^8 28^ Building on this and guided by the gendered hypothesis framework outlined by Parsons *et al*,^9^ we conducted theory-driven, exploratory snowball searches across relevant scientific databases such as PubMed/MEDLINE. Our search focused specifically on literature discussing gendered inequities in sport that could impact AE measurements. To enhance the depth of our analysis, the co-first authors each conducted a detailed review of key aspects: one focusing on training-to-match ratios and the other on team size, which informed our overall evaluation and recommendations.

### Equity, diversity and inclusion statement

The author group consists of members of the GenderSci Lab, a collaborative, interdisciplinary and international research lab dedicated to generating feminist concepts, methods and theories for scientific research on sex and gender and advancing the intersectional study of gender in the biomedical and allied sciences. The authors are all cisgender women and include two epidemiology doctoral students (ACD and AG), an associate professor in a Department for Health (SB) and a professor of the History and Philosophy of Science and of Studies of Women, Gender and Sexuality (SR).

## AEs and their limitations in the context of sports epidemiology

The construct of AE has emerged in the sports epidemiology literature as one of the most commonly used metrics to estimate exposure time. Prior to the advent of AEs, it was most common to calculate injury risk as the number of injuries per 100 athletes (*ie*, cumulative incidence or probability).^31^ AEs were introduced with the establishment of the National Athletic Injury/Illness Reporting System (NAIRS) surveillance database in the USA in 1975,^32 33^ with the proposition that calculating injury rate per AE would be a ‘more representative’ measure that considers ‘the frequency with which athletes are exposed to the potential of injury’.^34^ One AE was defined in NAIRS—and continues to be defined today—as an ‘opportunity for an athlete to get hurt’,^34^ calculated by ‘multiplying the average practice squad size times the number of practices […] and/or the average game squad size by the number of games’.^33^ AEs are also used as the measure of exposure in the US-based National Collegiate Athletic Association (NCAA) Injury Surveillance System (ISS), which was established in 1982.^35^ The NCAA ISS is the world’s largest sports injury database and serves as the data source for much of the evidence base on injury risk and prevention; to date, almost 200 studies have been published using this data,^36^ meaning that a large portion of the sports injury literature necessarily uses AEs. AEs are now also commonly used in studies of sports injury outside of these two surveillance databases and are considered to be ‘widely accepted’ and ‘a more accurate measure to define an injury’ than cumulative incidence.^37^

Despite their wide use, there are acknowledged limitations to the construct of AEs. Even at their inception, it was recognised that ‘more precise calculations of exposures are possible and often desirable for specialized investigations’, but it was deemed that ‘for surveillance purposes the athlete-exposure index generally suffices’.^34^ The most precise measure of exposure time would be the number of minutes or hours of sports participation for each individual athlete, which AEs do not capture.^26^ Rather, AEs are aggregate measures of exposure time at the group level and do not reflect the time that individual athletes actually spend exercising.^38^ The NCAA ISS database explicitly notes that ‘a reportable athlete-exposure was defined as one student-athlete participating in one practice or competition in which he or she was exposed to the possibility of athletic injury, regardless of the time associated with that participation,’ though players with zero playing time in a game are not included in that game’s exposure.^35^ Some studies, however, calculate participation in one practice or game by including all players on the game roster, whether they played or not.^2939^,^42^ AEs, therefore, give no indication as to the extent of participation of each player, which could range from continuous play throughout the match or practice, to a few minutes of play, to potentially no play. As such, AEs systematically overestimate athlete time at risk, with the magnitude of the bias dependent on the ratio of athletes actually playing at any given time to the number of athletes counted as ‘participating’ for the purposes of AE calculation.^29 43^ Indeed, a 2014 validation substudy comparing retrospectively calculated aggregate AEs to prospectively collected data on the number of athletes that actually participated in each practice and match found that retrospectively calculated AEs overestimated the team’s exposure by 27% for both men and women.^40^

In recognition of these challenges, a 2020 International Olympic Committee (IOC) consensus statement cautioned against collecting exposure data at the team level.^25^ Moreover, the assumption that AEs are comparable for athletes engaging in a particular sport at a specific level may also lack warrant.^38 40 44^ This aggregation across players is seen as justified because the volume of practices and competitions as well as the duration of the season is similar within sports categories and proficiency levels. However, an accurate investigation of risk factors for injury requires data on total AEs, injuries incurred and athlete characteristics to be collected for each individual player.^25 29^

## AEs in the context of gender/sex comparisons

### Training:match ratio

In conventional operationalisations of AEs, all AEs count equally towards the estimation of total exposure time, regardless of whether individual-unit AEs were accrued during training sessions or competitions. To compute injury rates, it is common practice to divide the total number of injuries that occurred during a given time period by the cumulative number of practice AEs and game AEs, without differentiating between the two. Empirical observations suggest, however, that the risk of injury is up to 10-fold higher during matches compared with training sessions.^3845^,^48^ This way of handling AEs translates, in effect, to the risk of injury being homogeneously distributed across the total time that athletes engage in physical activity, which is an inaccurate reflection of reality. When the goal of the analysis is to estimate injury risk in a restricted study population, this may be a valid approach because adding together training and competition AEs allows researchers to estimate total exposure time for that specific sample of athletes. However, aggregating training and competition AEs may not be suitable for comparing groups for which training-to-match (T:M) ratios systematically differ.

This consideration is particularly important in the context of researching gender/sex disparities in ACL injury, as women may have lower T:M ratios compared with men, even when playing at comparable proficiency levels and in similar contexts.^35 39 49^ That is, the proportion of time that women athletes spend training—as opposed to competing, when injury is more likely—may be lower than for men regardless of potential differences in competition volume. For example, women’s teams’ T:M ratio was 13% lower than that of men’s teams in two different studies of Swedish elite football players.^49 50^ Gender disparities in degrees of professionalisation and resource allocation, as well as in responsibilities outside of sports may account for differences in the amount of time that women athletes can devote to training in preparation for competitions.^51^,^54^ Optimal conditioning is key for preventing ACL injury, as both undertraining and overtraining are risk factors for injury.^55 56^ In this sense, lower T:M ratios among women may both make comparisons of injury rates by gender/sex using AEs invalid as an artefact of data and put women at factually higher risk of injury.

**Figure 1:**
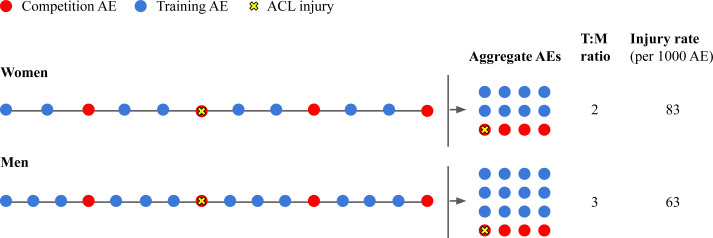
Lower T:M ratios among women may cause high-injury-risk AEs (competition AEs) to be over-represented in aggregate counts of AEs compared with men. ACL, anterior cruciate ligament; AE, athlete-exposure; T:M, training:match.

Despite the lack of studies quantifying the potential effect of T:M ratios on observed gender/sex disparities in ACL injury rates, gender differences in T:M ratios may contribute to an inflation of injury rates for women compared with those for men (figure 1). For example, an international meta-analysis of ACL injury among football players across all sporting levels found that the proportion of injured athletes (percentage of athletes who were injured) was the same for women and men, despite the injury rate (the number of injuries per unit of time) among women being twice as high as that among men.^27^ As illustrated in figure 1, this suggests that the lower rates of injury among men may be driven in part by the fact that their exposure time is often larger than for women, potentially due to higher training volume.

Studies comparing women’s and men’s teams within the same professional category show that the number of AEs accrued by men is systematically higher, on average, than that accrued by women across a variety of sports and countries,^8 27 28^ which may be due to differences in training volume as well as team size or number of teams included in an analysis. Salient disparities in training time can start from a young age. For example, one study found that among Finnish 15-year-old athletes, boys averaged 2.5 more hours of training per week compared with girls.^57^ At the collegiate level, an analysis of the NCAA ISS showed that women’s basketball and ice hockey teams had fewer practices per season compared with men on average, a difference that was most pronounced among Division III players (*eg*, 71 practices for men’s and 64 for women’s basketball, and 66 practices for men’s and 60 for women’s ice hockey).^35^ Notably, accounting for differing T:M ratios could also help contextualise the finding that gender/sex disparities in ACL injury are more pronounced among amateur-level athletes compared with professional or intermediate-level ones,^28^ as less professionalised women athletes may face significant barriers to spending time training, including childcare responsibilities and the need to work in other employment.^53^

Most studies examining gender/sex disparities in ACL injury report rates calculated without disaggregating training and competition AEs.^8 27 28^ Not accounting for potential gender/sex differences in T:M ratios jeopardises the validity of using aggregate measures of AEs to conduct comparisons between women and men. The use of AEs as a measure of exposure time seeks to standardise injury risk proportionally to the time that women and men spend doing physical activity. But a key assumption for comparability—that the distribution of risk over time is similar across the two groups—could be violated in this case, as the proportion of time at low risk of injury (*ie*, practices) versus the proportion of time at high risk of injury (*ie*, competitions) may differ between women and men. If women have a lower T:M ratio, competition-linked AEs will be over-represented in the cumulative count of their AEs compared with men’s. This is not captured when aggregate measures of AEs are used as a basis for comparison across genders and may lead to misleading conclusions regarding the aetiology of gender/sex disparities in injury.

### Number of players

While the higher number of AEs among men’s sports is likely driven in part by higher training volume, it could also be an artefact of systematic gender inequality in team size. Many women’s teams have smaller rosters than their corresponding men’s teams, likely stemming from systemic and persistent underinvestment in women’s sports. Expenditures on collegiate Division I men’s sports in the USA are double that of women’s sports, including substantial differences in resources for recruiting, scholarships and coaches’ salaries.^58^ Large differences in recruiting expenditures also occur in divisions II and III.^58^ Unsurprisingly, then, women’s college sport participation rate in the USA is lower than that of men: women made up 44% of NCAA student-athletes in 2020, despite accounting for 55% of enrolled students. Unbalanced participation by gender also occurs at the high school level, where girls similarly account for 43% of student-athletes.^58^ The gender pay gap in professional sports is well documented and is typically claimed to be due to the differences in revenue that men’s and women’s teams generate.^59 60^ However, the US women’s national soccer (football) team has more wins, higher viewership and generates more revenue than the men’s team, yet was still paid substantially less prior to recent legal battles for pay equity.^61^ Moreover, cultural misogyny and aversion to women’s sports as antithetical to hegemonic femininity likely play a role in shaping consumer preferences that lead to the higher revenue for most men’s sports.^62^

Given that AEs are often calculated by multiplying team size by the number of practices and matches, smaller roster size among some women’s teams would result in a smaller number of AEs, which could systematically overestimate the rate of injury among women compared with men. Data from a number of sources indicate that women’s team sizes tend to be smaller than men’s. The average NCAA Division I team size in 2023 was smaller among women than men in many sports according to the NCSA, a US collegiate sports recruiting platform (table 1).^63^ As an example, men’s association soccer (football) teams had an average of 31.7 players, compared with 30.4 players on women’s teams, despite the same number of athletes allowed in play (11). Professional football teams in the UK have similar imbalances; for example, the Arsenal men’s roster is 27 players compared with 24 players on their women’s roster,^64^ and Manchester United has 31 players on their men’s roster and 21 on the women’s.^65^ Moreover, men’s teams were allowed to increase their roster from 23 to 26 players for the 2022 FIFA Men’s World Cup, while women’s teams were still limited to 23 players for the Women’s World Cup in 2023.^66^ In the 2024 Olympics, although all ice hockey teams could only ‘dress’ a maximum of 20 skaters and 2 goaltenders for each game, men’s rosters were allowed up to 25 players and women’s rosters were allowed 23.^67^ In addition, although men’s lacrosse is played by 10 athletes per team on the field and women’s lacrosse is played by 12 athletes per team, average NCAA college lacrosse roster size is much larger for men (50.8) than women (34.3), indicating an even greater disproportionate inflation of AEs for men (table 1).

**Table 1:** Average NCAA Division I roster size in 2023^63^ and NCAA game and practice participants^35^

Sport	Average men’s roster size	Average women’s roster size	Average men’s game participants	Average women’s game participants	Average men’s practice participants	Average women’s practice participants
Basketball	16.0	14.5	10.5±1.9	10.2±1.8	14.3±3.5	12.3±2.7
Ice hockey	28.4	25.8	19.2±1.3	17.6±2.8	26.1±4.4	20.6±4.4
Soccer (football)	31.7	30.4	16.1±2.7	15.9±2.7	23.1±5.6	20.1±4.6
Lacrosse*	50.8	34.3	23.0±5.8	16.3±3.0	30.7±8.6	21.0±5.4

* Each sport in this table allows the same number of athletes on the playing field by gender apart from lacrosse, which is played by 10 men per team and 12 women women per team.

NCAANational Collegiate Athletic Association

**Figure 2:**
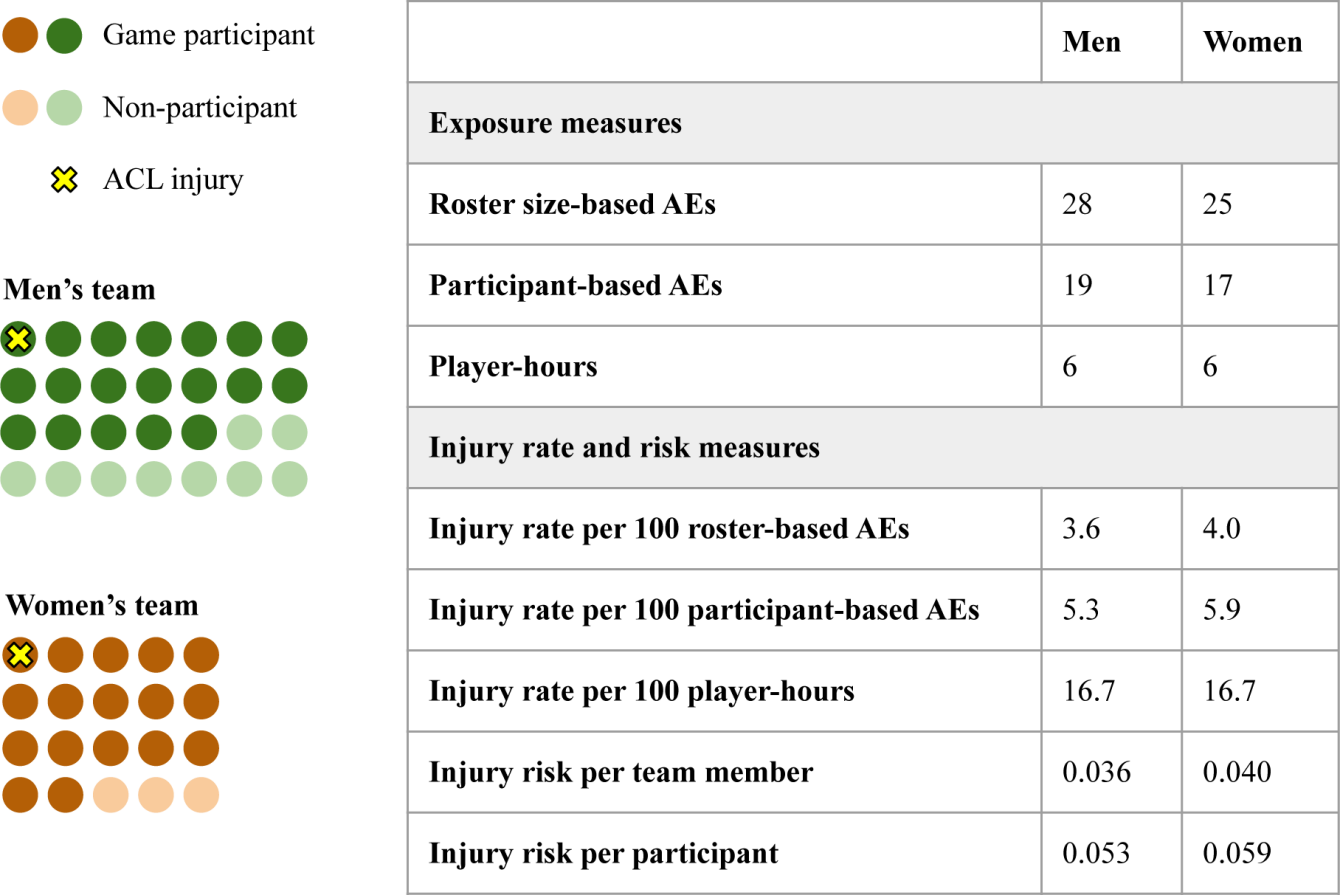
Example of the impact of men’s and women’s ice hockey roster size on calculated exposure time, injury rate, and injury risk. This figure represents one men’s and one women’s team participating in one 60-minute ice hockey match, in which six players per team are allowed on the ice at a given time and unlimited substitutions are allowed. See Box 2 for a narrative translation of this example and online supplemental figure 1 for further details on calculations. ACL, anterior cruciate ligament; AEs, athlete-exposures.

If each individual woman and man had comparable playing time in practices and matches despite differing team sizes, then the larger number of AEs for men’s teams would be artificially inflated and would misrepresent ACL injury rates when compared with those of women. However, even assuming practice time per player may be similar regardless of team size, larger men’s roster size likely means greater turnover during games for sports in which the number of players on the field during a match is the same for men and women and unlimited substitutions are allowed. Greater turnover would, in turn, result in a lower individual time at risk for most men compared with women. This is corroborated by data from the NCAA ISS demonstrating a higher average number of game participants in men’s ice hockey (19.2±1.3) than in women’s ice hockey (17.6±2.8), despite the fact that the number of players allowed to be on ice at any given time is the same. Time at risk during matches is particularly important, as ACL injury risk is much higher during matches compared with training.^47 68 69^ This means that the risk (rather than the rate) of injury for each female athlete may be higher than that of each male athlete, due to greater match play time. AEs can, therefore, both overestimate the rate of injury per unit time in women compared with men and obscure the higher probability of injury per individual woman athlete (figure 2 and box 2), both of which are driven by structural, gendered inequities in team size, rather than innate biological factors.

Box 2 **Narrative example: how gender differences in team roster size could affect injury rates calculated using AEs**

Consider a 60 min ice hockey game, which is played by six players per team at a given time, despite different average team sizes and participants per game for women and men (figure 2). Assuming no overtime play, there are six player-hours of time at risk for injury for a team in a single match. If there was one ACL injury in this given game, the injury rate per player-hour would be the same for men and women (16.7 injuries per 100 player-hours). However, if AEs are calculated by multiplying the number of game participants by the number of games, we would expect to calculate approximately 19 AEs for men and 17 AEs for women for a single ice hockey game (given an average of 19.2 and 17.6 game participants in men’s and women’s ice hockey, respectively; see table 1). This method of exposure classification would result in an injury rate of 5.3 ACL injuries per 100 AEs for men and 5.9 per 100 AEs for women, despite the exact same number of injuries and the exact same number of total athlete-minutes of gameplay. If AEs were calculated by multiplying the total roster size by the number of games, the time at risk would also be inflated for men, resulting in an artificially deflated injury rate for men: 3.6 injuries per 100 AEs for men, 4.0 per 100 AEs for women. However, at the individual level, time at risk would be approximately 19 min for each men’s ice hockey player and 21 min for each women’s player. If we again assume one injury occurred in each game, each participating man has a 5.3% probability and each woman has a 5.9% probability of ACL injury risk per game. Therefore, despite the same rate of injury per minutes of gameplay by gender, probability of injury in an individual athlete over the course of a single game or over a season is slightly higher among women, simply because smaller team size means women athletes play for a higher proportion of each game.

ACL, anterior cruciate ligament; AEs, athlete-exposures.

## Contextualising AEs and observed gender/sex disparities in ACL injury rates: recommendations

AEs can be a useful tool in understanding injury risk. In order to make these a better measure of exposure time for the purposes of comparing injury rates across genders, we offer the following five recommendations:

1. Acknowledge the limitations of AEs as a construct of exposure time.
2. Record training and match time separately, as recommended by the IOC consensus statement,^25^ and adjust for T:M ratio in statistical analyses.
3. Use more accurate measures of total time at risk such as the sum of individual-level player hours, as recommended by the IOC^25^ or analytically control for team size.
4. Develop and implement methods for collecting more nuanced data on AEs, including considerations of the quality and type of training and the sport environment.
5. Contextualise observed gender/sex disparities in injury rates with discussion of gendered differences in training quality, environment, resource allocation and history.^9^

Ultimately, understanding gender/sex disparities in injury risk requires considering a number of gendered environmental factors that athlete exposures do not and cannot capture. When comparing injury risk across groups sampled from different contexts and from different underlying populations, researchers must consider whether the AEs that comprise the rate denominator for each group are not only quantitatively, but also qualitatively comparable, in terms of type and level of training, facilities and equipment, among other factors. As such, in addition to understanding and quantifying exposure, we urge researchers to layer or triangulate understandings of AEs with a gendered environmental approach using qualitative methods.^9^

Qualitative approaches can help illuminate important gender differences in the quality of training or match exposure. Girls’ and women’s sport suffers from systematic underinvestment, resulting in the environments in which they participate and compete being sub-par when compared with those of boys and men.^9^ Further, the quantity and quality of the training itself—key factors for preventing ACL injury—are known to be lower, too.^70^ In this way, historically rooted systemic inequities between women’s and men’s sports continue to shape sport practices and cultures, including the resources available to teams, compensation structures for athletes, and—consequently—not only the amount of time that athletes can devote to training in preparation for competitions, but the calibre of that training, too.^9 71 72^ This is further compounded by lower quality facilities (*eg*, competitions occurring on artificial rather than natural turf; lack of access to high-quality gyms and training equipment) and training load potentially being much higher in a shorter time period (*eg*, smaller team roster sizes and congested tournament schedules).^73^,^77^ These considerations are particularly important in the context of researching gender/sex disparities in ACL injury. In addition, male practice players are sometimes used in high-level women’s sports, which may further complicate comparisons of injury rates by gender/sex using AEs. While this practice can help reduce fatigue by allowing women athletes greater opportunity to rest, it may also differentially increase training load for starting players by making training more intense.

The limitations of the construct of AE for appropriately ascertaining the existence, extent and drivers of gender/sex disparities in ACL injuries underscores a need for a greater base of empirical evidence capturing the influence of gendered exposures to physical activity and sport over the lifecourse, with particular attention to training age. Gendered disparities at every level of participation in sport may play a significant role in shaping the cumulative qualitative experience and volume of athlete exposures. This will aid understanding how gendered exposures emerge, as well as the impacts of these exposures.

## Conclusion

It is crucial to account for the gendered context in which public health metrics arise in order to accurately interpret observed gender/sex differences, as has been demonstrated for outcomes ranging from COVID-19^78^ to adverse drug events.^79^ Despite their wide circulation and use, AEs are a worryingly undertheorised and underscrutinised construct for comparisons across genders. No attention has been devoted to examining how the assumptions underlying the operationalisation of AEs as a measure of exposure time in research may affect estimates of gender/sex disparities in ACL injury, and whether AEs offer, in fact, a valid basis on which to conduct comparisons by gender. Acknowledging these assumptions, recognising the limitations, and developing and implementing methods for capturing and accounting for nuances (such as T:M ratio, team size and the quality of training), will ultimately generate more valid and robust statistical inference on injury risk by gender/sex. Such approaches will, we hold, ultimately reveal important gendered, contextual factors in injury risk that have the potential to shape injury prevention research for ACL as well as other injuries.

## supplementary material

10.1136/bjsports-2024-108812 online supplemental figure 1
