# Supplementary material for: Limitations of athlete-exposures as a construct for comparisons of injury rates by gender/sex: a narrative review
Source: Br J Sports Med. 2024 Dec 4;59(3):e108812. doi: 10.1136/bjsports-2024-108812 (PMC11874318; doi:10.1136/bjsports-2024-108812)
Supplement: online supplemental figure 1 [file bjsports-59-3-s001.pdf]

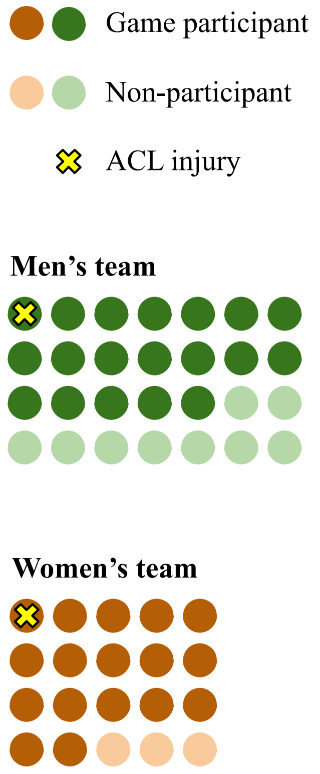

	Men	Women
**Exposure measures**		
**Roster size-based AEs**	28 players × 1 game<br>= **28**	25 players × 1 game<br>= **25**
**Participant-based AEs**	19 participants × 1 game<br>= **19**	17 participants × 1 game<br>= **17**
**Player-hours**	6 participants × 1 hour game<br>= **6**	6 participants × 1 hour game<br>= **6**
**Injury rate and risk measures**		
**Injury rate per 100 roster-based AEs**	1 injury ÷ 28 AEs × 100<br>= **3.6**	1 injury ÷ 25 AEs × 100<br>= **4.0**
**Injury rate per 100 participant-based AEs**	1 injury ÷ 19 AEs × 100<br>= **5.3**	1 injury ÷ 17 AEs × 100<br>= **5.9**
**Injury rate per 100 player-hours**	1 injury ÷ 6 player-hours × 100<br>= **16.7**	1 injury ÷ 6 player-hours × 100<br>=**16.7**
**Injury risk per team member**	1 injury ÷ 28 players<br>= **0.036**	1 injury ÷ 25 players<br>= **0.040**
**Injury risk per participant**	1 injury ÷ 19 participants<br>= **0.053**	1 injury ÷ 17 participants<br>= **0.059**
